# Large-Scale and Comprehensive Immune Profiling and Functional Analysis of Normal Human Aging

**DOI:** 10.1371/journal.pone.0133627

**Published:** 2015-07-21

**Authors:** Chan C. Whiting, Janet Siebert, Aaron M. Newman, Hong-wu Du, Ash A. Alizadeh, Jorg Goronzy, Cornelia M. Weyand, Eswar Krishnan, C. Garrison Fathman, Holden T. Maecker

**Affiliations:** 1 Institute for Immunity, Transplantation, and Infection, Stanford School of Medicine, Stanford University, Stanford, CA, United States of America; 2 Division of Rheumatology and Immunology, Department of Medicine, Stanford University School of Medicine, Stanford, CA, United States of America; 3 CytoAnalytics, Denver, CO, United States of America; 4 Division of Oncology, Department of Medicine, Stanford University School of Medicine, Stanford, CA, United States of America; Jackson Laboratory, UNITED STATES

## Abstract

While many age-associated immune changes have been reported, a comprehensive set of metrics of immune aging is lacking. Here we report data from 243 healthy adults aged 40–97, for whom we measured clinical and functional parameters, serum cytokines, cytokines and gene expression in stimulated and unstimulated PBMC, PBMC phenotypes, and cytokine-stimulated pSTAT signaling in whole blood. Although highly heterogeneous across individuals, many of these assays revealed trends by age, sex, and CMV status, to greater or lesser degrees. Age, then sex and CMV status, showed the greatest impact on the immune system, as measured by the percentage of assay readouts with significant differences. An elastic net regression model could optimally predict age with 14 analytes from different assays. This reinforces the importance of multivariate analysis for defining a healthy immune system. These data provide a reference for others measuring immune parameters in older people.

## Introduction

Many changes have been reported in the aging human immune system, or implicated from work on aging mice. One of the earliest observations of immune aging was decreased antibody production in response to vaccination or other challenge, including germinal center dysfunction and decreased somatic hypermutation [1,2]. It is also well-appreciated that naïve T cell numbers decline with age, as a result of decreased thymic output [3]. Selective reductions in hematopoietic stem cells committed to lymphoid development further reduce the pool of naïve lymphocytes [4,5], and this in particular reduces the number of naïve B cells with age [6].

A loss in naïve B and T cell numbers can contribute to defects in the acquired immune response, simply as a result of loss in antigen receptor diversity [7]. However, there are also many age-associated functional defects reported on the cellular level. These seem to be mostly associated with naïve cells, as memory T cell functions appear to be largely preserved throughout life [8]. A number of naïve CD4+ and CD8+ T cell signaling defects were recently reported in a study of pSTAT signaling in aging [9]. Defects in the Erk signaling pathway, mediated at least in part by increased DUSP4 activity in the elderly, have also been reported [10].

Perhaps as a result of signaling defects, along with the influence of repeated antigen encounter, a trend towards cellular immune senescence in the elderly has been described [11]. This is evidenced by progressive loss of costimulatory receptors such as CD28 on CD8+ T cells, with concomitant increases in regulatory receptors or markers of exhaustion [12,13]. While the phenotype of aged and antigen-exhausted T cells show some differences [11], it is tempting to speculate that replicative senescence is a consequence of the long-term expansion of effector T cells to chronic antigens, in combination with a lack of naïve T cell replacements.

Clearly, other factors besides age play into the development of terminally differentiated and/or exhausted T cells; chief among these is CMV exposure. The CMV response in otherwise healthy individuals is large and dominated by terminally differentiated (CD28-CD27-CD45RA+) CD8+ T cells [14–16]. Indeed, the entire CD8+ T cell compartment is skewed toward more mature and terminally differentiated cells in individuals with long-term CMV exposure [17]. CMV infection has further been shown to reduce telomere lengths in circulating T cells [18], as well as to increase CD8 clonality [19]. These are all phenomena typically associated with aging. However, it is possible to dissect at least some effects of age from those of CMV infection alone [7,20].

Receptors for sex hormones can be found on various immune cells, so sex would also be expected to influence the composition and function of the aging immune system. Indeed, significant differences in certain serum cytokine levels between males and females have been described [21–23]. Sex as well as age differences have also been noted in response to immunogenic cancers such as melanoma [24,25].

In the current study, we sought to create a resource of immune measurements in aging adults, with specific attention to the influence of age, sex, and CMV status on the immune system. Rather than test specific hypotheses, we aimed to develop a database from which metrics of immunological health could be gleaned. We chose assays to comprehensively examine the immune system at the level of secreted proteins, cells, and gene expression. These included assays based on stimulation with cytokines (for phospho-epitope analysis) or with a cocktail of LPS, IFNα, CD3+CD28 beads, and anti-IgM+IgG (for cytokine secretion and gene expression analysis). Our results probe the independent and combined effects of age, sex, and CMV on multiple readouts of immune phenotype and function. We use multivariate approaches to examine relationships between these readouts, and to build a model of the most significant predictors of age. However, we fully expect our data to be mined in many additional ways, and have therefore made the entire data set available through ImmPort (SDY420, http://immport.niaid.nih.gov), with a visual overview of analyzed immune parameters available at http://earlybird.cytoanalytics.com.

## Results

### Clinical Demographics of the Healthy Aging Cohort

**Fig 1:**
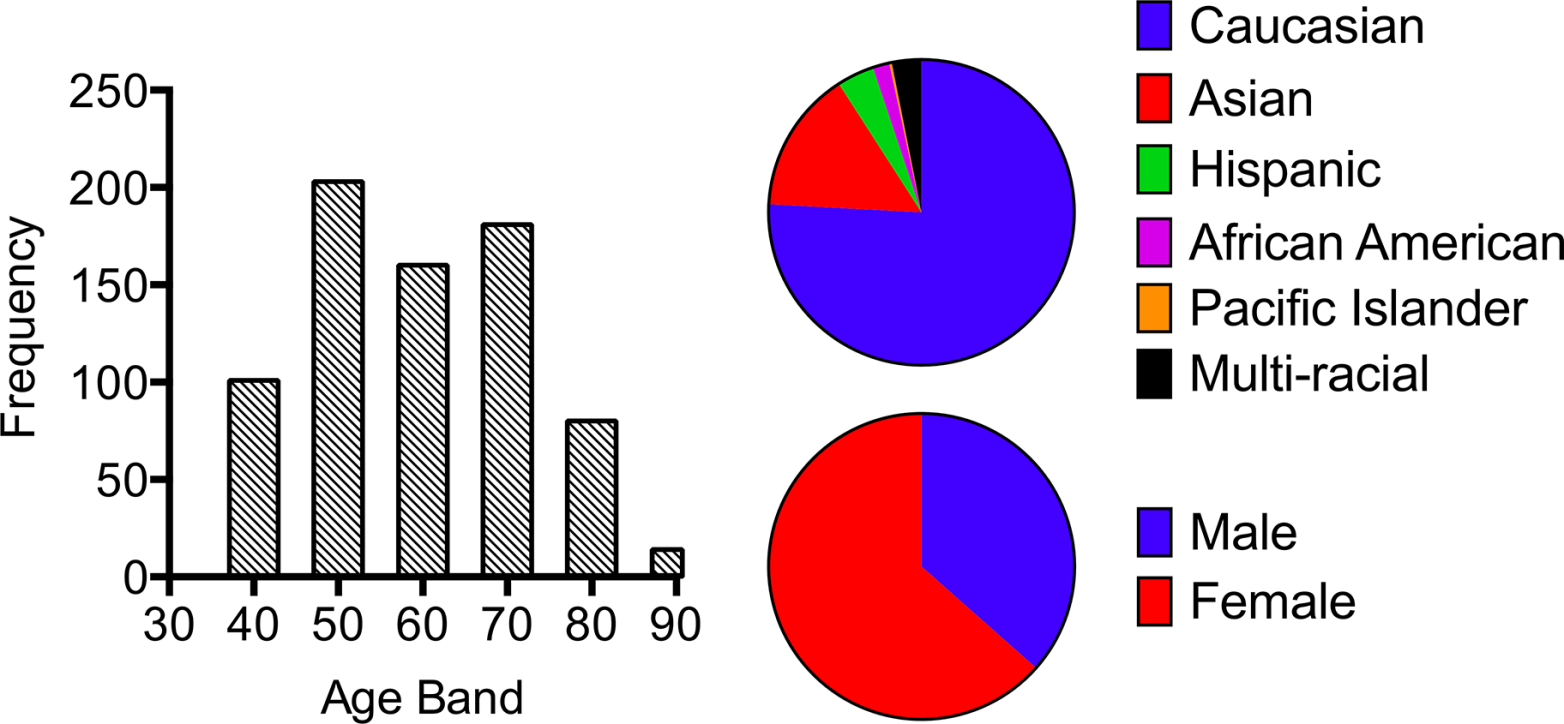
Demographics of the cohort of 740 participants, including age band, race/ethnicity, and sex. Results are representative of the aging population in the San Francisco Bay Area. A subset of 240 of these individuals was selected for further immunological analysis; this subset included all individuals age 78 and above, with an equal number of 40–50 year olds, and lower representation of the middle age bands.

We recruited 740 individuals aged 40–97, in and around Palo Alto, California. To avoid the confounders of overt disease, a defined list of exclusion criteria was developed (Table 1). Participants were enrolled to ensure age and sex balance, and the resulting cohort approximately mirrored San Francisco Bay Area ethnic demographics (Fig 1). Participants completed a set of functional assessments and clinical laboratory tests, as described in the Methods. Additional blood was used for our specialized immunological tests and for banking of serum, PBMC, RNA, and DNA.

**Table 1:** Study inclusion and exclusion criteria.

**Inclusion criteria**
Age 40+ years
Ability to informed consent
Ability to complete questionnaires
Willingness to be followed up over several years.
**Exclusion criteria**
Cancer (except skin cancer)
Any infection in the past 3 months
Active allergy or hay fever
HIV
Hepatitis A, B, C
Organ transplant recipient
Autoimmune diseases such as Rheumatoid Arthritis, SLE, Multiple Sclerosis
Uncontrolled diabetes or thyroid problems
Stroke/ heart attack in the last 1 month
Cirrhosis of liver
Active herpes of mouth or genitals
Alcoholism
Active Psoriasis; use of steroid creams in the past 4 weeks.
Prescription anti-inflammatories, anti-allergy, antibiotic or steroid use in the past 3 months
Lifetime use of Chemotherapy especially cytotoxic agents.
Alcohol consumption more than 2 cans of beer/2 shots of whiskey/2 glasses of wine daily in the past 30 days


### Summary of Significant Associations with Age, Sex, and CMV Status

We completed comprehensive immunophenotyping and functional analyses on 243 subjects, chosen to include a continuum of all age groups, but with emphasis on the youngest and oldest individuals (all subjects age 78 and above were included, balanced by an equal number of 40–50 year olds). This selection allowed us the greatest likelihood of finding immunological differences with age. The assays applied to this subset included immunophenotyping by mass cytometry (CyTOF); phospho-epitope flow cytometry; gene expression analysis on unstimulated and stimulated PBMCs; and Luminex cytokine assays on the supernatants of those PBMC, as well as on serum. PBMC stimulation was done with a cocktail of IFNα, LPS, CD3+CD28 beads, and anti-IgM+IgG antibodies. These stimuli were chosen to trigger largely mutually exclusive signaling pathways in immune cells (see PBMC Stimulation Assays in the Methods).

**Fig 2:**
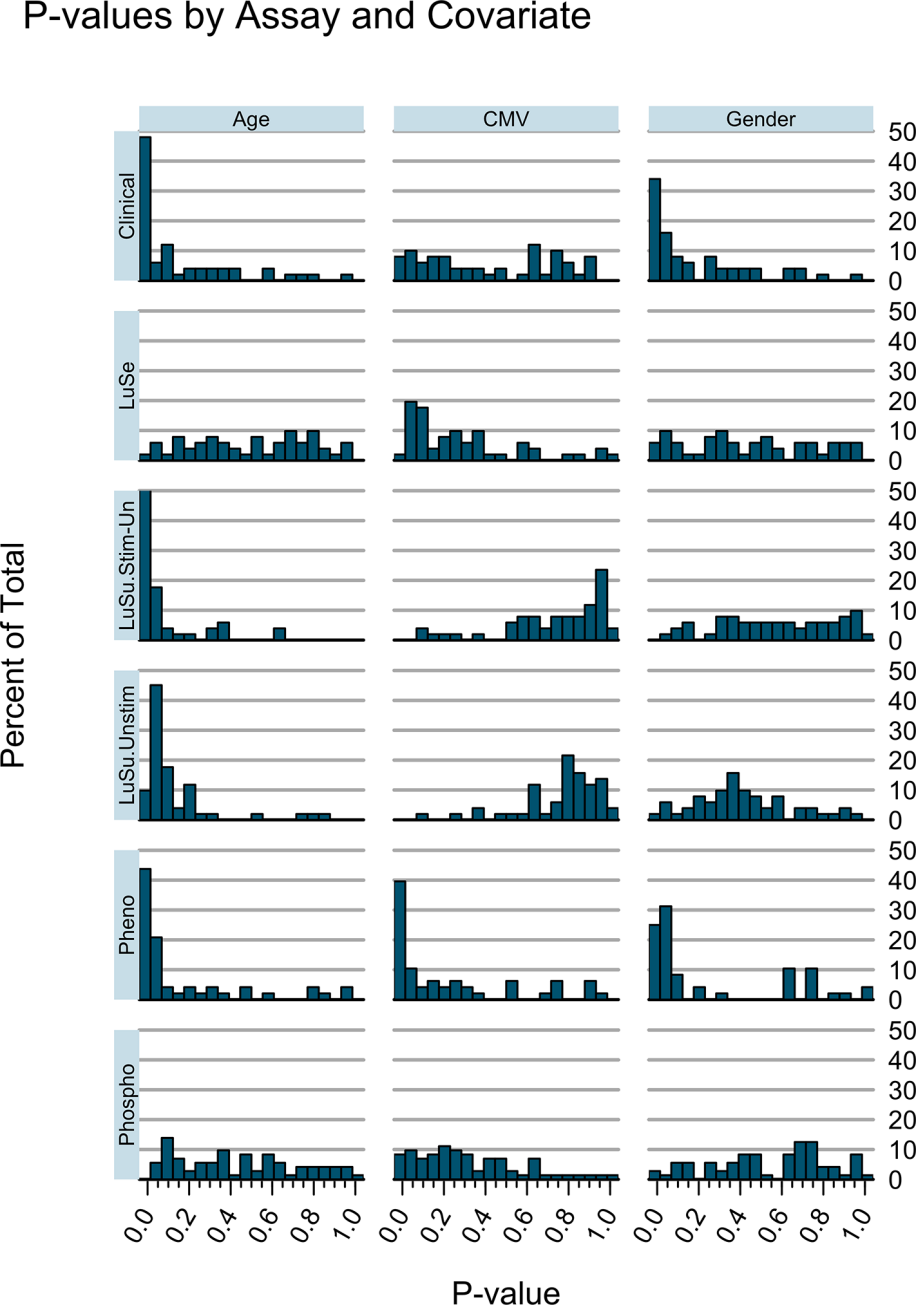
Overview of age, sex, and CMV-status associations with laboratory and clinical assay results. Histograms show the distribution of P values for the analytes within each assay category. Clinical = all clinical variables; LuSe = serum Luminex; LuSuptStim-Un = PBMC supernatant Luminex, Stimulated-Unstimulated values; LuSuptUnstim = PBMC supernatant Luminex, Unstimulated values only; Pheno = CyTOF immune phenotyping; PhopshoFC = phospho-flow cytometry. It is apparent that the clinical variables yield low P values primarily for age and sex. Stimulated Luminex assays yield more low P values than serum Luminex assays, and those P values are largely restricted to age. Flow cytometry phenotyping reveals a higher proportion of sex and CMV effects, while phospho-flow cytometry did not yield many low P values for any covariate (age, sex, or CMV status).

An overview of the combined analysis results from these different assays is shown in Fig 2. Here the percentage of assay readouts with particular P values for age, CMV status, or sex is graphed as a histogram. From this summary, it is apparent that different assays revealed age, CMV, and sex differences to varying degrees. For example, a high proportion of readouts for the clinical, stimulated/unstimulated Luminex, and immunophenotyping assays had P values <0.05; but this was not the case for serum Luminex or phospho-flow. Immunophenotyping appeared to be most influenced by CMV status, while immunophenotyping and clinical assays had the most significant changes with sex.

Another way to gauge the output of these assays is to examine the readouts with the lowest P values for each assay (Table 2). Here, clinical laboratory tests and functional assessments are both included as separate “assays”. While this compilation ignores the fact that some assays may have many more significant readouts than others, it highlights what each assay is “best” at. For example, phospho-flow uniquely identified only CMV differences among the five readouts with lowest P value. Immunophenotyping identified age and CMV differences among its “top five”, while the other assays identified a combination of mostly age and sex differences. A complete list of all readouts with P values for age, sex, and CMV status is provided in S1 Table.

**Table 2:** Five analytes with lowest unadjusted P value for age, sex, or CMV status, by assay or assay group.

Assay	Analyte	Covariate	Covariate Coefficient^1^	P
Hematology and blood chemistry	hemo	Sex	1.432	1.00E-21
	hema	Sex	3.8713	4.80E-19
	ureanitrog	Age	0.1634	5.60E-15
	rbc	Sex	0.3761	1.70E-12
	creatinine	Sex	0.1909	3.40E-12
Functional assessment	grip_r	Sex	14.8652	3.10E-41
	grip_r	Age	-0.2933	1.10E-19
	promisscore	Age	0.0111	5.00E-10
	wk8ft_time	Age	0.0536	8.10E-10
	pe_wc	Sex	3.5334	4.00E-09
Serum cytokines	LEPTIN	Sex	-0.7837	1.40E-10
	MIG	Age	0.0245	1.30E-09
	ENA78	Sex	-0.428	9.50E-04
	RANTES	Sex	-0.3667	5.00E-03
	IFNA	CMV	0.3424	1.10E-02
PBMC cytokines (Stimulated-Unstimulated)	MCP3	Age	-0.0322	4.20E-12
	ENA78	Age	-0.0271	3.80E-10
	IL7	Age	-0.0187	1.90E-08
	ICAM1	Age	-0.0185	9.00E-08
	VCAM1	Age	-0.0212	1.80E-07
PBMC cytokines (Unstimulated only)	MCP3	Age	0.0151	6.00E-05
	VCAM1	Age	0.0123	2.20E-04
	ENA78	Age	0.0094	1.30E-03
	MCP3	Sex	0.3084	6.80E-03
	IL15	Age	0.0072	8.00E-03
Immunophenotyping	CD27+CD8+ T cells	CMV	-20.6371	1.80E-18
	naive CD8+ T cells	Age	-0.5944	1.40E-14
	CD94+CD8+ T cells	CMV	15.4194	1.70E-09
	CD8+ T cells	CMV	9.1306	5.80E-09
	B cells	Age	-0.3764	7.50E-09
Phospho-epitope flow cytometry	CD8+: pSTAT3.IFNa	CMV	-0.3306	3.80E-04
	CD8+: pSTAT1.IFNa	CMV	-0.2367	1.10E-03
	CD8+: pSTAT3.IL-6	CMV	-0.4443	1.10E-03
	CD4+: pSTAT3.IFNa	CMV	-0.288	4.60E-03
	CD8+: pSTAT5.IFNa	CMV	-0.1774	5.90E-03

^1^ “Covariate Coefficient” is the estimated value for the adjustment associated with the indicated covariate. For Age, the coefficient indicates the estimated amount by which the analyte increases (or decreases, if negative) for each year of age. For CMV, the coefficient indicates the estimated amount by which the analyte increases (or decreases) for study participants who are CMV positive. For Sex, the coefficient indicates the estimated amount by which the analyte increases (or decreases) for study participants who are male. Units of measure are specific to the assay or analyte.

**Fig 3:**
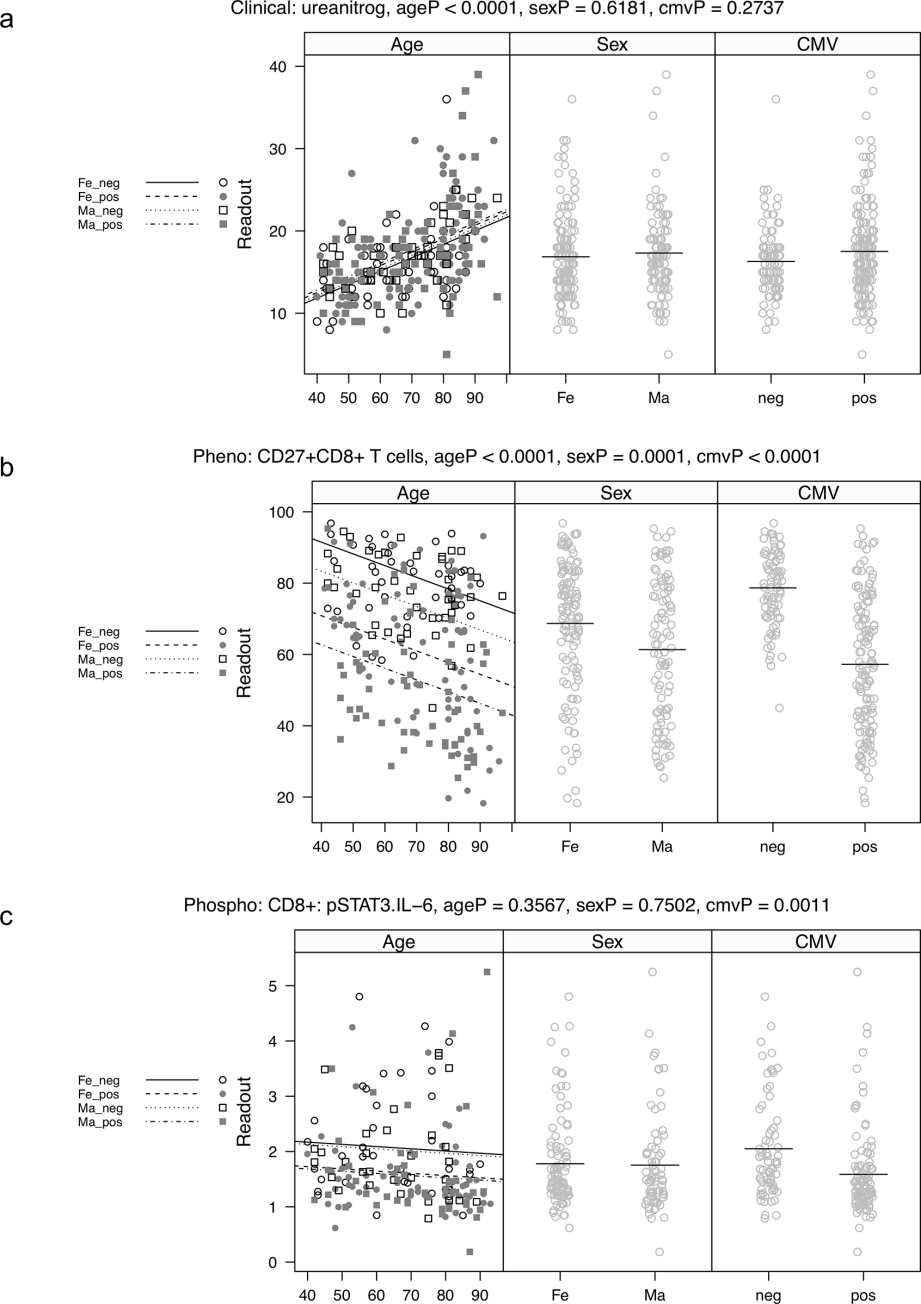
(A) Example of a clinical laboratory assay, urea nitrogen, which shows a significant correlation with age (unadjusted P value = 5.8x10^-15), but not with sex or CMV status. All p-values are unadjusted p-values from a multiple linear regression as described in the methods. The p-values test the significance of the indicated predictor variable, while adjusting for the other two variables. The left most panel (age) shows age versus urea nitrogen, with fitted regression lines for CMV negative females (Fe_neg, solid), CMV positive females (Fe_pos, dash), CMV negative males (Ma_neg, dot), and CMV positive males (Ma_pos, dot dash). Observations from each of these four groups are indicated by different symbols. The second panel (Sex) shows observations by sex, with horizontal reference lines at group means. The third panel (CMV) shows observations by CMV status, with horizontal reference lines at group means. (B) Example of a cell subset (CD27+CD8+ T cells) that has significant correlations with age, sex, and CMV status. The Y axis represents the percentage of CD27+CD8+ T cells within all CD8+ T cells. The CMV effect is the most dramatic (unadjusted P value = 1.7x10^-18). (C) Example of a phospho-flow cytometry readout (IL-6 stimulated pSTAT3 in CD8+ T cells) that has a significant CMV effect (P = .0035), but no significant age or sex relationship. The Y axis represents the fold change in pSTAT3 in CD8+ T cells, unstimulated/IL-6 stimulated.

Examples of significant readouts from each assay or assay group are shown in Fig 3. Fig 3A shows the relationship of a clinical laboratory test, urea nitrogen, with age, sex, and CMV status. This analyte showed a significant relationship with age, but not sex or CMV status. By contrast, Fig 3B shows associations for an immunophenotyping analyte, CD27+CD8+ T cells. This analyte showed a highly significant relationship with age, sex, and CMV status. Finally, Fig 3C shows these associations for a phospho-flow analyte, IL-6-stimulated pSTAT3 in CD8+ T cells. Here, the only significant relationship appears to be with CMV status. These examples further demonstrate the different “strengths” of each assay in identifying significant relationships with age, sex, or CMV.

### Cytokines in Serum versus Stimulated PBMC

**Fig 4:**
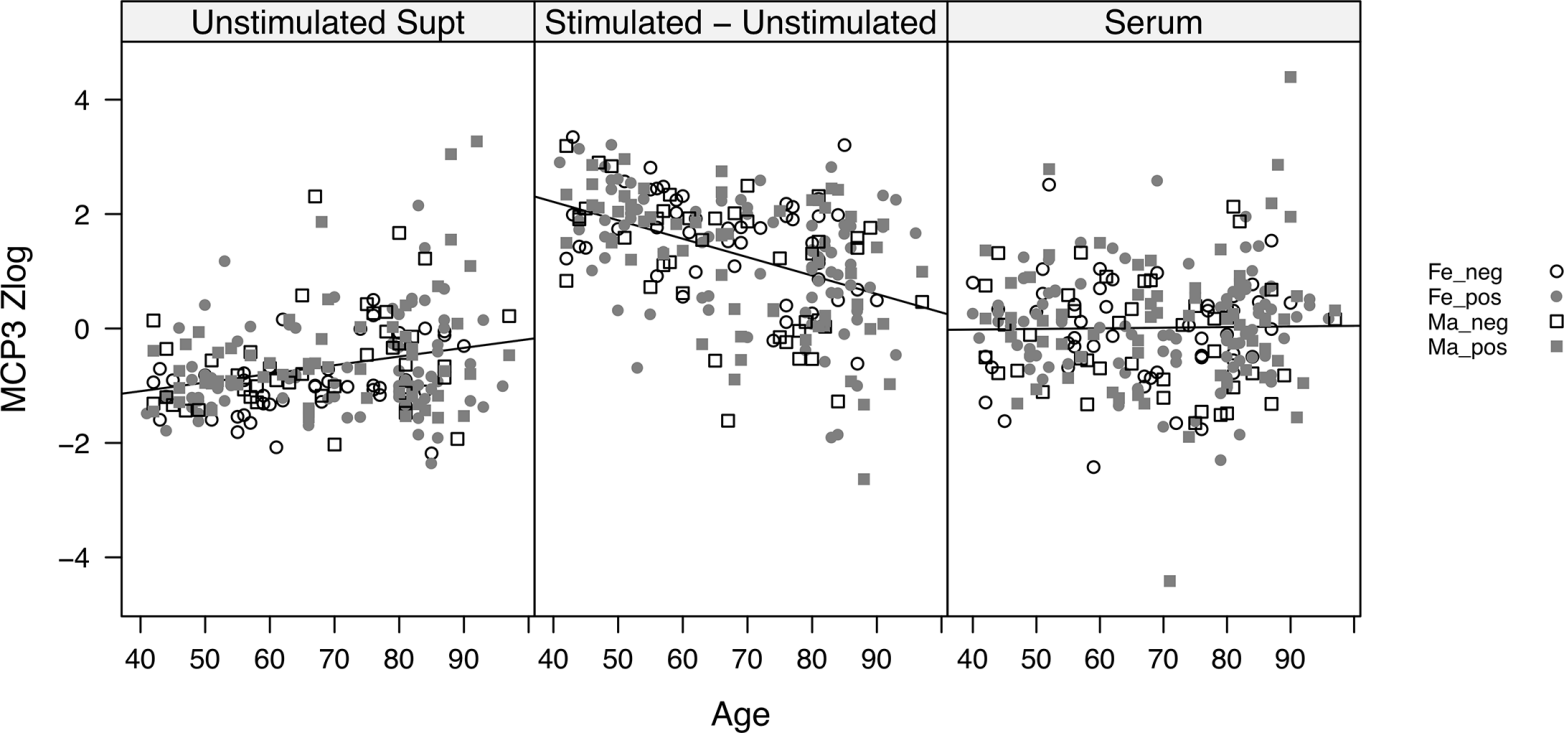
Example of a cytokine, MCP-3 (also known as CCL7) that shows differing trends with age depending upon the type of assay. Left panel, supernatant levels from unstimulated PBMC incubated for 4 hours. Middle panel, supernatant levels from PBMC stimulated with a cocktail of IFNα, LPS, CD3+CD28, and anti-IgM+IgG, after subtraction of unstimulated supernatant levels. Right panel, serum levels. All cytokines were measured by a 63-plex Luminex panel. Y-axis values represent the z score of median fluorescence intensity (MFI), after log2 transformation. Black line shows fitted regressions of analyte readout versus age. While CMV effects were not significant in any assay, age effects were significant for both unstimulated and stimulated-unstimulated supernatant assays.

An interesting comparison is the differing age associations of cytokines when measured in serum versus stimulated PBMC supernatants. Many cytokines display a pattern similar to that shown for MCP3 in Fig 4. When analyzed in the supernatant of PBMC incubated without stimulation, this cytokine shows a slight upward trend with age (driven in part by some high outliers among the oldest individuals). However, when stimulated with a cocktail of IFNα, LPS, CD3+CD28, and anti-IgM+IgG, a strong downward trend with age emerges (after subtraction of the unstimulated levels). Finally, when MCP3 levels are analyzed in serum, there is no significant trend with age. Thus, a requirement for *in vitro* activation of cells in order to uncover functional differences with age is revealed in this example.

### Stimulated Gene Expression

**Fig 5:**
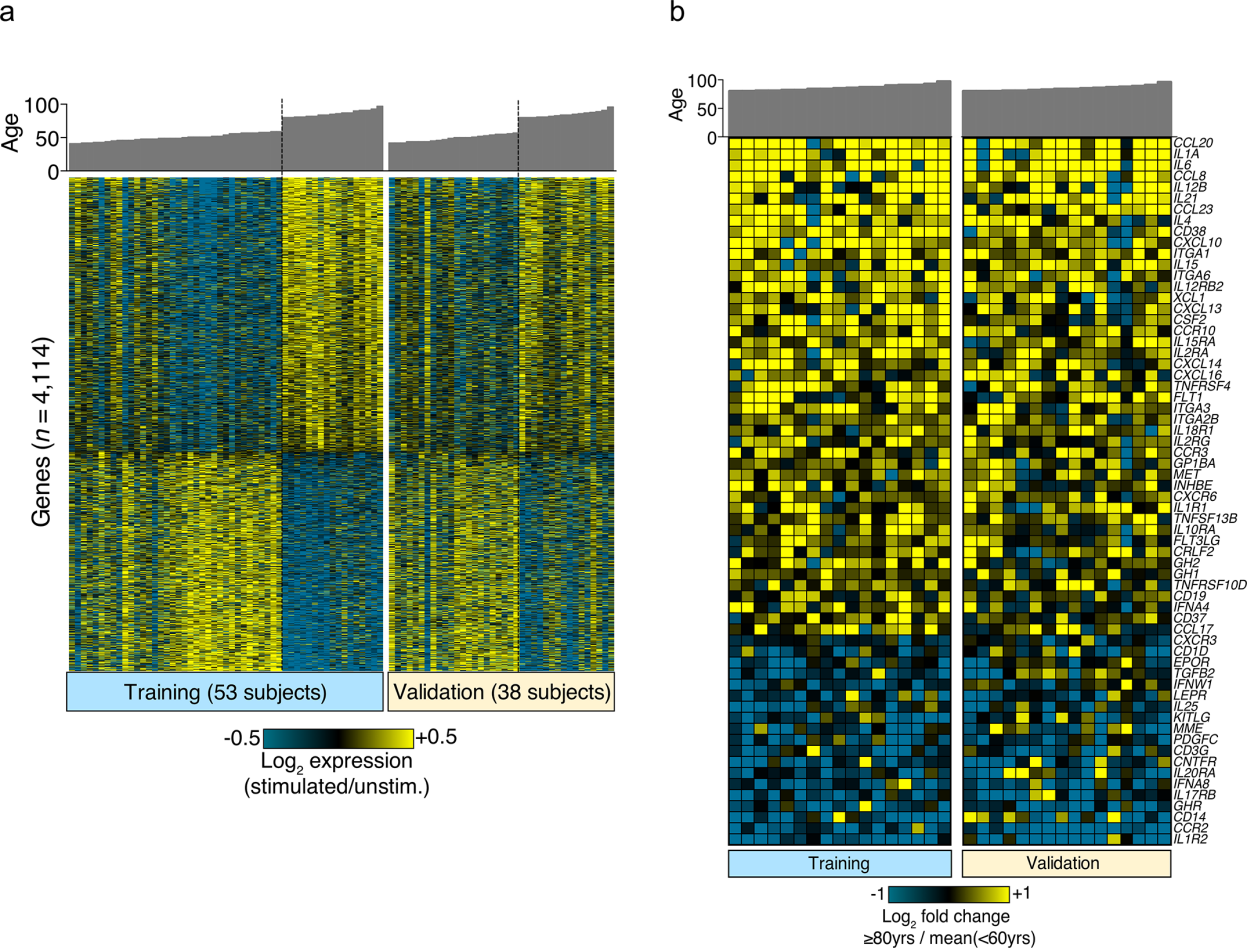
Differentially expressed genes by age in stimulated PBMC. After 4 hours incubation of PBMC with or without a cocktail of IFNα, LPS, CD3+CD28, and anti-IgM+IgG, RNA was extracted and used for gene expression microarray analysis (Agilent, 4x44K, 2-color). Subjects <60 y and > = 80 y were randomly divided into a training set (53 subjects) and validation set (38 subjects). 4114 genes were identified in the training set as differentially expressed in the older vs. younger subset. (A) Differentially expressed genes are ordered by decreasing fold change between older and younger groups in the training set (left panel). A similar segregation is seen in the validation group (right panel). In these panels, expression values are represented as log2 expression(stimulated/unstimulated). (B) Display of 64 immune-related genes from the 4114 gene signature. In these panels, expression values are represented as fold change compared to the mean level of each gene in the young group. While many genes were associated with age, no gene signature was found to be specifically associated with CMV or sex (data not shown).

For analysis of gene expression in stimulated PBMC, we analyzed a subset of younger (<60 y; n = 58) and older (> = 80 y; n = 33) individuals, and randomly split them into internal training and validation groups in a ~3:2 ratio, respectively. Of the 20,000 genes probed in the arrays, we identified 4114 significantly expressed genes that were age-associated (Fig 5A). 2128 genes were down-regulated and 2286 were up-regulated with age (S2 Table). A further analysis of 64 immune-related genes is shown in Fig 5B. All of these genes are present in the immune-related KEGG pathways falling either in the cytokine-receptor interaction or hematopoetic cell system. The top canonical pathways were “role of pattern recognition receptors in recognition of bacteria and viruses” and “role of cytokines in mediating communication between immune cells”. The top regulators were IFNγ, TLR3, and LPS (S1 File). Consistent with previously reported observations, our data revealed a number of inflammatory cytokine genes with 2-fold or greater up-regulation in older versus younger individuals. These included IL-6, IL-1A, IL-21, IL-12B, IL-4, IL-2 as well as the chemokines CCL20, CCL23, and CCL8. Strikingly, a large number of up-regulated genes with enhanced expression are involved in immune response modulation by IFNγ, TLR3, LPS, TLR4, IRF7, IFNα2, TREM1, IRF1, IRF8, TCR and STAT1 (S1 File). While genes with higher or lower expression in older individuals were fairly balanced (Fig 5A), there was a tendency towards a more elevated transcriptional signature for immune genes in elderly individuals (Fig 5B).

### Multianalyte Visualization

**Fig 6:**
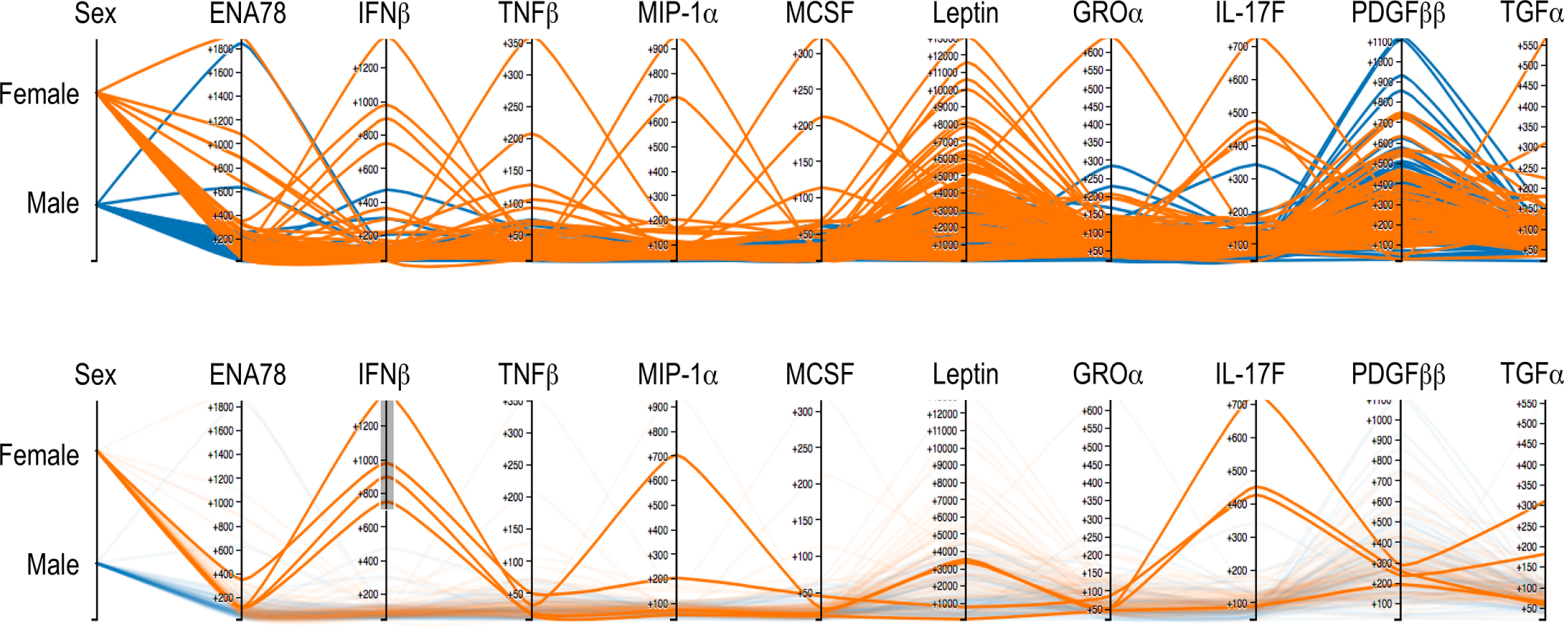
Example of multivariate visualization using parallel coordinates (http://earlybird.cytoanalytics.com). Results for the ten serum cytokines with highest variability are shown, with individuals colored by sex. Each analyte is displayed on its own vertical axis, with the axis range corresponding to the data range. Each line represents one person, and intersects each axis at the appropriate value (MFI) for that person. With the exception of PDGFββ, it is clear that the highest outliers are all females. By selectively highlighting the top individuals for IFNβ (bottom panel), it becomes apparent that many of these individuals are “serial outliers”, i.e., high for other cytokines as well. This implies a minority phenotype of females with multiple high serum cytokine levels.

In analyzing such a comprehensive data set, it is of course interesting to examine relationships among multiple analytes, rather than single analytes in isolation. This process is aided by visualizations such as the parallel coordinates view shown in Fig 6. In this Figure, the top ten cytokines with highest variance in serum were chosen for display, with individuals colored by sex. From the top panel, it is apparent that a relatively small number of high outliers account for most of the variance of these cytokines. Furthermore, these high outliers are almost exclusively females (except for PDGFββ, known to be higher in males [23]). By selecting those outliers that are high for one cytokine (IFNβ, bottom panel), we see that these IFNβ^high^ individuals are also high outliers for at least some of the other most variable cytokines. Thus, there are patterns of high expression of multiple cytokines, found exclusively in females, and revealed by the parallel coordinates visualization tool (see http://earlybird.cytoanalytics.com).

### Multivariate Modeling

**Fig 7:**
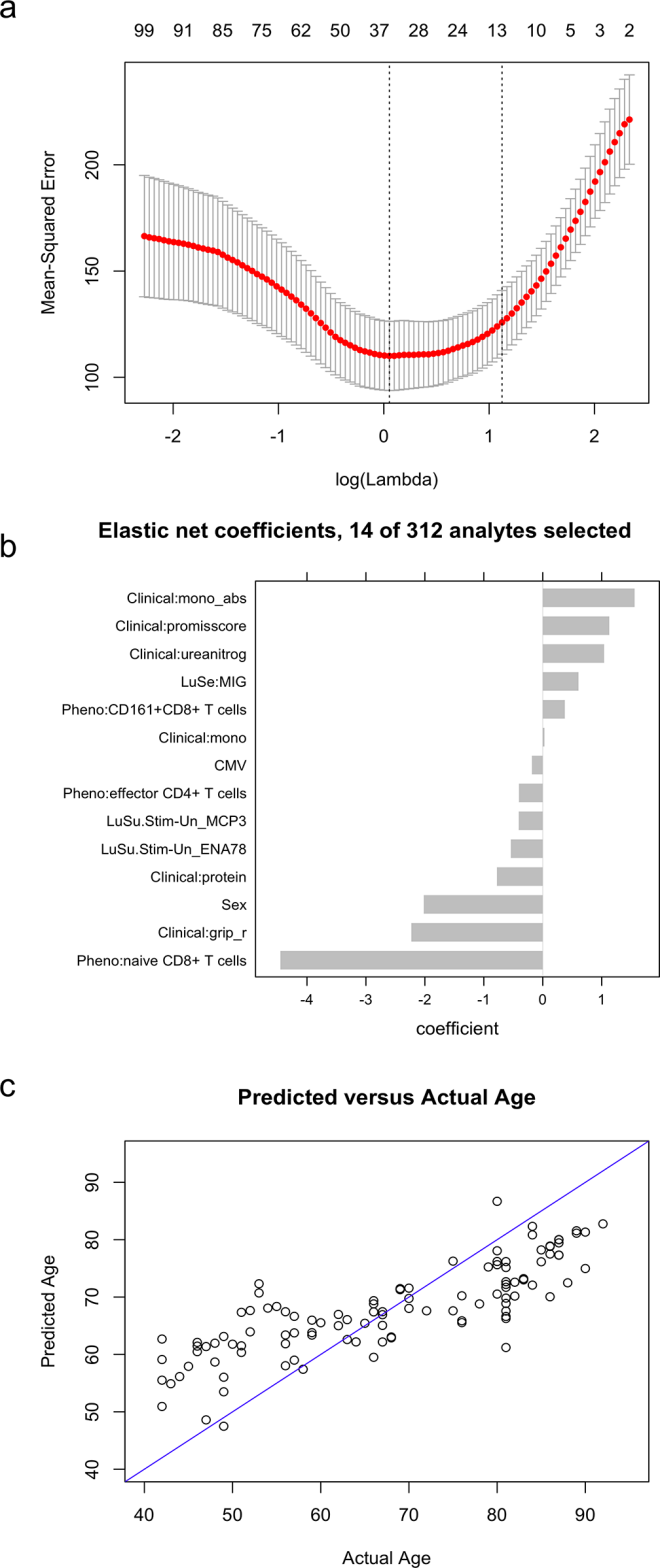
(A) Profile of mean-squared error of elastic-net regression for various values of lambda. For a range of lambdas, an elastic-net regression is fit using 10-fold cross-validation. Vertical dotted lines indicate the value of lambda for which MSE is minimized, and the largest value of lambda within one standard error of that minimum. The numbers along the top border of the graph indicate the number of non-zero parameters that are included in the model for corresponding values of lambda. (B) Results of elastic-net regression combining data from multiple assays. Each bar represents the coefficients for a particular predictor of age. Predictors are selected by the algorithm, with 14 out of 312 analytes chosen. Analytes from multiple assays (clinical, serum Luminex, flow phenotyping, and the stimulated PBMC supernatant Luminex) were selected. Sex and CMV status were explicitly included in the model. Predictors were converted to Z-scores prior to model fitting so that all analytes could be interpreted on a common scale. Each unit represents one standard deviation for that particular analyte. Coefficients represent adjustment to a mean age of 69, based on the scaled value of the analyte. Thus, for example, the estimated age increases by approximately 1.6 years for every unit increase in absolute monocyte count (the analyte with the largest positive coefficient), holding all other values constant; and decreases by approximately 4.5 years for every unit increase in naïve CD8+ T cells (the analyte with the largest negative coefficient). (C) Comparison of predicted versus actual age for the elastic-net model. Each point represents one person. The blue diagonal line represents predicted age = actual age. Interestingly, the resulting model overestimates the age of younger participants, and underestimates the age of older participants.

We further sought to build a model for predicting age that included analytes from multiple assays, We used the elastic net regression method [26], which estimates a linear relationship minimizing mean squared error (MSE) while using a small number of predictors that are uncorrelated with each other. The number of predictors is related to lambda, a term which penalizes the inclusion of additional predictors. The larger the lambda, the smaller the number of predictors. Lambda is usually selected to be the largest value within one standard error of the lambda which minimizes MSE. This is illustrated in Fig 7A, which also illustrates the relative performance of the various models. The 14-parameter model, with resulting coefficients for the chosen parameters, is shown in Fig 7B. Interestingly, of the 14 analytes chosen by the model, 6 are clinical laboratory or morphometric tests, and 3 are immunophenotyping subsets. The remainder include cytokines, sex, and CMV status. Of note, we did not include gene expression analytes, in order to avoid overwhelming the model and to keep it focused on immunological predictors. The fact that the immunological assays contribute at some level to the model suggests that they have some degree of prediction for age. A measure of performance of the model is shown in Fig 7C, which plots actual versus predicted age. It is apparent that the model tends to overestimate the age of younger individuals and underestimate the age of older individuals.

We also built elastic-net logistic regression models for sex and CMV status. Plots of lambda, coefficients for the chosen analytes, and performance versus true sex and CMV status are shown in S1 and S2 Figs. While the sex prediction model achieved nearly 100% accuracy, the CMV prediction model was slightly less accurate. While the CMV model was heavily dependent on immunophenotyping analytes, the sex prediction model contained a mixture of analytes from clinical, immunophenotyping, and other assays.

### Cross-Assay Associations

**Fig 8:**
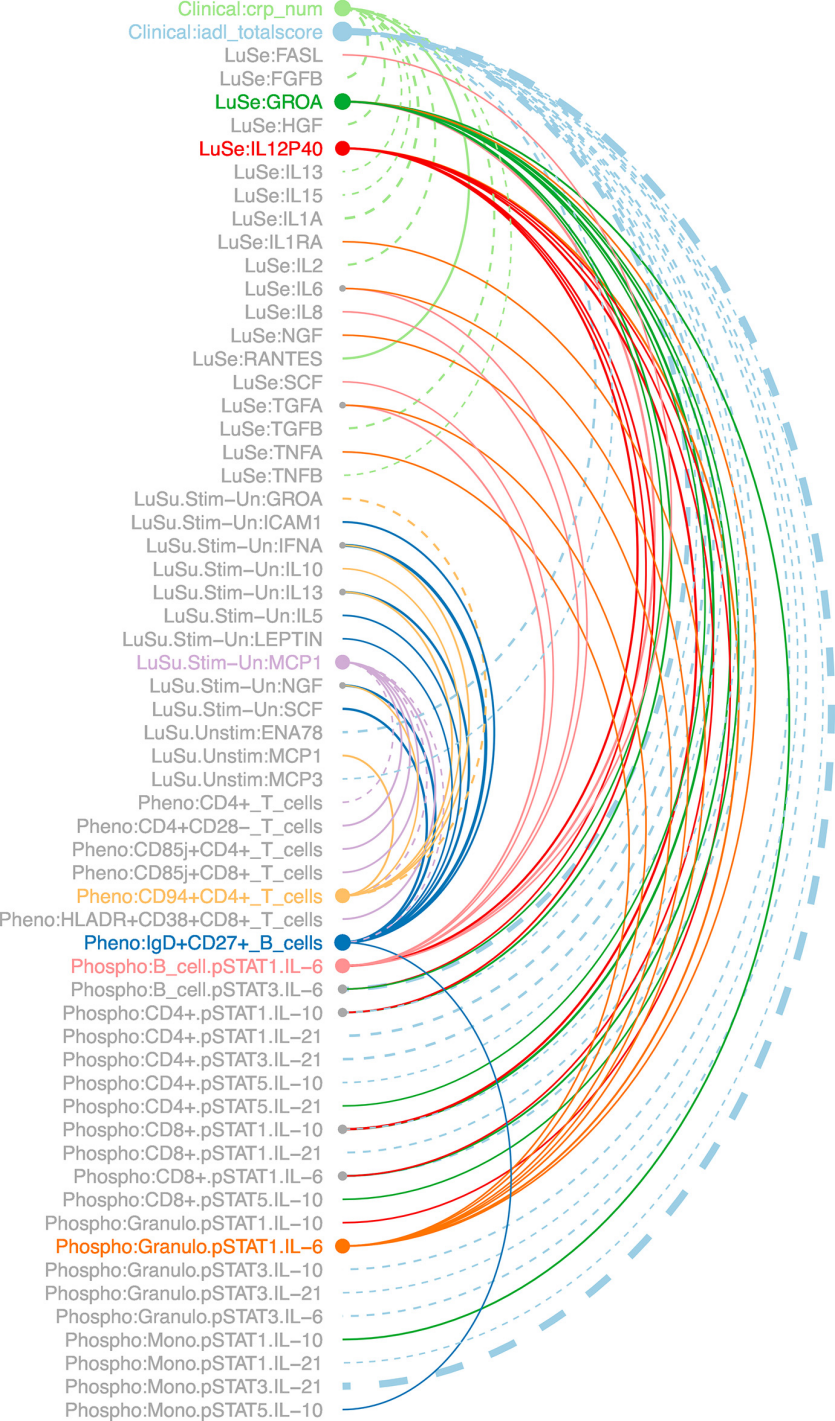
Associations between clinical and immunological analytes, after accounting for the effects of age, sex, and CMV status. Initially, associations were limited to the strongest 0.5% of the cross-assay associations, as defined by smallest p-values. The 3 Luminex assays were considered a single assay. We then looked for analytes having the largest numbers of associations, hereafter called connections. The diagram includes all analytes connected to at least 7 other analytes (n = 9), and the analytes to which these analytes are connected, for a total of 61 analytes. Node size is proportional to the number of connections. Solid lines represent positive correlation, dotted lines negative. Line width is proportional to absolute magnitude of correlation, with thicker lines representing stronger correlations. Line color differs for each of the 9 highly connected analytes.

To further explore cross-assay associations, we looked for significant associations between pairs of analytes, after accounting for the effects of age, sex, and CMV status. We then focused on statistically significant associations in which the two analytes were from different assays, with the 3 Luminex assays considered a single assay. We then identified the strongest 0.5% of the associations (n = 160, spanning 120 analytes), as defined by smallest p-values. P-values ranged from 2.6x10^-16^ (waist circumference association with serum leptin) to 1.0x10^-3^ (IL-10 in PBMC supernatant and CD94+CD4+ T cells). The largest effect size (0.71, measured in standard deviations) was for the association between waist circumference and leptin, suggesting that after accounting for age, gender, and CMV status, waist circumference increased by an average of 0.71 standard deviations for each SD increase in serum leptin. Details are provided in S4 Table. Analytes that appeared in the largest number of pairwise associations included the total score of the Instrumental Activities of Daily Living Scale (n = 15), C-reactive protein (n = 9), serum GroA (n = 9), and IgD+CD27+ B cells (n = 9). Fig 8 illustrates these connections. It includes 9 highly connected analytes having at least 7 associations as described above, and the analytes to which these primary analytes are connected. A total of 61 analytes are shown. This data suggests a high degree of relatedness between immunological readouts, especially between different types of assays, and also between clinical/morphometric measurements and immune assays.

## Discussion

In this study, we describe a large immunological data set based on about 240 individuals from a clinical cohort of 740 healthy aging adults. Data from cellular, protein, and genomic assays are described, with particular emphasis on stimulation-response assays (phosphoepitope analysis of cytokine signaling, and cytokine production and gene expression from stimulated PBMC). Our emphasis in this paper was to describe the features of each assay with regard to discovering differences based on age, sex, and CMV status. Further analysis of the data is welcomed, via a parallel coordinates visualization tool (http://earlybird.cytoanalytics.com), or via download from ImmPort (SDY420, http://immport.niaid.nih.gov).

One common theme among nearly all our immunological readouts is that there is considerable heterogeneity at every age, which is generally greater than the mean change across ages. This is exemplified by the CD27+CD8+ T cell subset (Fig 3B). While the downward trend with age is highly significant, the breadth of values at every age is very high. In this regard, there are essentially no “clock” analytes, which would accurately and independently predict age, since there is so much overlap in the distributions among young and old individuals. The corollary to this is that many, if not most, elderly individuals still fall within the range of the younger adults. In fact, we often observed a broadening of the distribution with age. This is exemplified by the cytokine MCP3 (Fig 4), for which high or low outliers are seen at increasing frequency with age, for all three assay methodologies; and by urea nitrogen (Fig 3A), for which high outliers are seen among the oldest participants. Trends of this type lead to an encouraging hypothesis, namely, that these outliers among the elderly might predict those at risk for immunologically-related diseases, and could perhaps provide diagnostic markers for immune intervention among the elderly. In this regard, it is important to note that this study had strict exclusion criteria (Table 1), such that overt diseases of aging were not clinically present. Although our data provide a relatively clean description of the range of immunological values associated with healthy aging, they represent a cross sectional analysis and as such may include individuals at risk for or developing disease.

By performing a series of assays at the cellular, protein, and genomic level, we were able to compare how the different assays performed with regard to identifying differences in age and other variables. We chose sex and CMV status as major covariates, due to the plethora of existing literature on immunological differences associated with them. Trends by age, sex, and CMV status were captured by all the immune assays, to greater or lesser degrees. For example, phospho-flow was most strongly associated with CMV status (Table 2), while stimulated cytokine production showed strong associations with age (Table 2, Fig 2). CyTOF phenotyping was associated with both age and CMV status (Table 2, Fig 2). Thus, in order to best quantify the effects of all three of these covariables, a comprehensive set of immune assays is most informative.

CMV serostatus was used as an endpoint for the model of S2 Fig This was because we were most interested in defining immune differences between those with CMV infection and those without. However, CMV IgG titers are included in our online data (www.immport.niaid.nih.gov), so one could also choose to model immune predictors of CMV antibody titer. Of note, we did not measure CMV viral load, which is generally undetectable in healthy donors, even those with apparently frequent reactivation [27].

As a result of quantifying significant readouts by age, sex, and CMV status, one can pose the question, “Which of these three variables has the greatest impact on the immune system as we measured it?” From the summary of P values in Fig 2, it seems reasonable to conclude that age, then sex and CMV status, show the greatest effects. Taken another way, it is impressive to see that the changes in the immune system brought about by a single pathogen, CMV, rival the differences seen between the sexes, in terms of the number of significantly affected analytes. Of course, the presence of statistically significant measurements should not be confused with biologically relevant changes, which are more difficult to quantify.

Another conclusion from our data is that there is a clear interaction of age, sex, and CMV status. For example, cell subsets (like CD27+CD8+ T cells, Fig 3B) are highly influenced by all three. In this case, the effect of CMV appears to be a downward broadening of the distribution, such that a subset of CMV+ individuals shows markedly lower levels of these cells. However, females and younger individuals have, on average, higher levels of these cells, so that a younger female with CMV is likely to have more CD27+CD8+ T cells than an older male with CMV. When all the immunological and clinical readouts were used to create an age prediction model, a series of 28 analytes spanning all the assays emerged. Thus, the most accurate prediction of age is achieved when data from multiple assays are combined.

The associations shown in Fig 8 and S4 Table also highlight the connectedness between different immune assays and between the immune system and clinical and morphometric tests. For example, we were struck by the high number of associations of immune readouts with an index of daily living activities. These types of associations can be taken as further evidence of the strong influence of environment on the immune system, as recently highlighted in a study of immunological characteristics of twins [28].

The interaction of multiple variables on the immune system, along with the heterogeneity seen in any single analyte among healthy individuals, suggests another important conclusion. Clinicians are accustomed to reference ranges for laboratory tests, with clear upper and lower limits, beyond which lies “abnormal”. But for most measurements in the immune system, the reference range for any one analyte is both wide and possibly dependent upon extrinsic factors, like sex, CMV status, and/or other covariables that may or may not be measured. In fact, given the data presented in Fig 6, the “normal” range for cytokines may in fact be dependent on the levels of other cytokines in that individual. This complicates the definition of normal immune parameters; but reinforces the importance of a highly comprehensive data set such as this one, when making comparisons to specific disease states.

## Materials and Methods

### Study Participant Recruitment and Measurements

740 participants, aged 40–97, were recruited in and around Palo Alto, CA. Exclusion criteria were modeled on The Baltimore Aging Longitudinal Study (http://www.blsa.nih.gov). We excluded individuals with common and clinically identifiable conditions that could directly affect the immune system (such as infections in the preceding 3 months and cancer in the past 2 years), or who were on medications that have such an effect (glucocorticoids, antihistamines). A complete list of inclusion/exclusion criteria is shown in Table 1. Non-institutionalized study volunteers aged 40 years and over who were in self determined good health were invited to contact the study center by phone, using traditional methods of recruitment such as local newspaper advertisements, flyers and brochures. Respondents underwent a brief telephone screening to determine eligibility. Those interested and eligible to participate were scheduled for a visit with a study coordinator. This visit was scheduled such that there was at least an 8-hour fast prior to blood sample collection. A consent form was mailed in advance of the visit, and the signed copy was collected during the visit. We enrolled participants in such a way that age-gender numerical balance was achieved.

Study-related assessments included a specific questionnaire, phlebotomy and selected functional tests listed below. The consent packet included HIPPAA authorization for access to medical records and the consent allows re-contact of participants for future research visits. The key components of the study visits were: consent process, a patient-administered review of systems, medical and immunization history (corroborated by review of medical records), a Health Assessment Questionnaire [29], and Godin Leisure-time physical activity questionnaire [30]. Participants 65 years in age or older also underwent additional evaluation for Frailty using 3 metrics: (a) the 7-point Clinical Frailty Scale [31], (b) Cardiovascular health Study Frailty index [32], and (c) Rockwood Criteria [31]. Phlebotomy was performed on fasting individuals and a 55 mL blood sample was obtained. The blood sample was transported to the Human Immune Monitoring Center (HIMC) laboratory for processing and storage. A small portion was sent for clinical laboratory testing including a complete blood count, ESR, high resolution C-reactive protein, a comprehensive metabolic panel, a fasting lipid panel and CMV serology.

The study was approved by the Stanford University Institutional Review Board for the Protection of Human Subjects (Stanford IRB). All participants provided written informed consent and the study was performed under the supervision of the Stanford IRB.

### Luminex Assays

This and all of the following assays were performed in the Human Immune Monitoring Center at Stanford University. Human 51-plex kits were purchased from Affymetrix (Santa Clara, CA) and used according to the manufacturer’s recommendations with modifications as described below. Briefly, samples were mixed with antibody-linked polystyrene beads on 96-well filter-bottom plates and incubated at room temperature for 2 h followed by overnight incubation at 4°C. Room temperature incubation steps were performed on an orbital shaker at 500–600 rpm. Plates were vacuum filtered and washed twice with wash buffer, then incubated with biotinylated detection antibody for 2 h at room temperature. Samples were then filtered and washed twice as above and re-suspended in streptavidin-PE. After incubation for 40 minutes at room temperature, two additional vacuum washes were performed, and the samples re-suspended in Reading Buffer. Each sample was measured in duplicate. Plates were read using a Luminex 200 instrument with a lower bound of 100 beads per sample per cytokine. Custom assay control beads by Radix Biosolutions (Georgetown, TX) were added to all wells.

### CyTOF Immunophenotyping

PBMCs were thawed in warm media, washed twice, resuspended in CyFACS buffer (PBS supplemented with 2% BSA, 2 mM EDTA, and 0.1% soium azide), and viable cells were counted by Vicell. Cells were added to a V-bottom microtiter plate at 1.5 million viable cells/well and washed once by pelleting and resuspension in fresh CyFACS buffer. The cells were stained for 60 min on ice with 50 uL of the following antibody-polymer conjugate cocktail: [insert Ab list here]. All antibodies were from purified unconjugated, carrier-protein-free stocks from BD Biosciences, Biolegend, or R&D Systems. The polymer and metal isotopes were from DVS Sciences. The cells were washed twice by pelleting and resuspension with 250 uL FACS buffer. The cells were resuspended in 100 uL PBS buffer containing 2 ug/mL Live-Dead (DOTA-maleimide (Macrocyclics) containing natural-abundance indium). The cells were washed twice by pelleting and resuspension with 250 uL PBS. The cells were resuspended in 100 uL 2% PFA in PBS and placed at 4C overnight. The next day, the cells were pelleted and washed by resuspension in fresh PBS. The cells were resuspended in 100 uL eBiosciences permeabilization buffer (1x in PBS) and placed on ice for 45 min before washing twice with 250 uL PBS. If intracellular staining was performed, the cells were resuspended in 50 uL antibody cocktail in CyFACS for 1 hour on ice before washing twice in CyFACS. The cells were resuspended in 100 uL iridium-containing DNA intercalator (1:2000 dilution in PBS; DVS Sciences) and incubated at room temperature for 20 min. The cells were washed twice in 250 uL MilliQ water. The cells were diluted in a total volume of 700 uL in MilliQ water before injection into the CyTOF (DVS/Fluidigm, Toronto, Canada). Data analysis was performed using FlowJo v9.3 (CyTOF settings) by gating on intact cells based on the iridium isotopes from the intercalator, then on singlets by Ir191 vs cell length, then on live cells (Indium-LiveDead minus population), followed by cell subset-specific gating.

### PhosphoFlow Staining

Smart Tubes (Smart Tube Inc., Palo Alto, CA) containing 10,000 U IFNα, or 50 ng IL-6, IL-10, or IL-21, were incubated with 1 ml/tube of heparinized whole blood within two hours of draw. After 15 min at 37C, Proteomic Stabizer was automatically added by the Smart Tube base station, and samples were cooled to 4C, followed by freezing at -80C.

Prior to thawing samples, PBS (Phosphate Buffered Saline), FACS Buffer (PBS+0.5% BSA), and 1X Thaw Lyse Buffer 2 (Smart Tube, Inc.; diluted from 5X concentrate with ddH2O) were pre-warmed to room temperature. Smart Tubes were thawed in a 10‐12°C water bath for 25 minutes. Approximately 0.25 ml of the contents of each Smart Tube were put through a cell strainer (~70 micron mesh) into a 15 ml conical tube, washed with 12 ml of 1X Thaw‐Lyse Buffer, and incubated at room temperature for 10 minutes. Cells were then pelleted at 600 x g for 5 minutes at room temperature. Supernatant was discarded and the pellet resuspended in Thaw‐Lyse Buffer again, followed by centrifugation at 600 x g for 5 minutes at room temperature. Supernatant was discarded and the pellet was resuspended in 0.5 ml PBS and transferred to a deep well plate and washed once with PBS. The sample was then stained with the following cell surface marker panel for 30 minutes at room temperature: PerCP cy5.5 CD66b (Biolegend 305108), PE‐Cy7 CD33 (BD 333946), Pac Blue CD3 (BD 558117), Ax 700 CD4 (BD557922), AX 700 CD19 (BD557921), Pac Blue CD14 (BD558121). After washing with FACS buffer, cells were permeabilized by adding 600μl cold MeOH to each well of the deep well block using a multichannel pipette, and mixing by pipetting up and down in each well. After further washing, the different stimulus conditions were barcoded using a 3x3 matrix with Pacific Orange and Alexa Fluor 750 (Invitrogen Corp.) at 0.03 and 0.04 mg/ml for low and 0.2 and 0.3 mg/ml for high staining, respectively. Incubation with barcoding dyes was performed at 4C for 30 min. After several washes with FACS buffer, stimulated and barcoded cells were pooled into single tubes and stained for 30 min at 4C with the following intracellular antibody panel: Ax488 pSTAT‐1 10 (BD 612596), Ax647 pSTAT‐3 (BD557815), PE pSTAT‐5 (BD612567). After two further washes with FACS buffer, samples were resuspended in PBS and acquired on an LSRII flow cytometer (BD Biosciences). Data was analyzed with FlowJo software (TreeStar).

### PBMC Stimulation Assay

We analyzed both gene expression and secreted cytokines from a PBMC stimulation assay. For this we chose a cocktail of LPS, IFNα, CD3+CD28 beads, and anti-IgM+IgG antibodies, to interrogate TLR, cytokine, T cell receptor, and immunoglobulin receptor signaling, respectively. We initially showed that the genes responsive to these individual stimuli were largely independent after 4 h stimulation, resulting in mostly additive results when using the stimulation cocktail. Furthermore, gene expression changes generally tracked well with changes in the corresponding protein by Luminex.

To perform this assay, PBMC were thawed in media in a 37°C water bath then 1 ml of warm media containing 20 uL benzonase (1:10000, 25U/ml) was slowly was slowly added. The cells were transferred to a 15ml centrifuge tube (with 8 ml of warm media), then another 1ml of warm media was added to retrieve all cells. Cells were centrifuged at 490 g for 8 mins at room temperature, supernatant removed, and the pellet resuspended in 1ml warmed benzonase media by tapping the tube. 9 ml warmed media were then added to the tube, and the cells centrifuged at 490 g for 8 mins at room temperature. Supernatant was decanted, and cells again resuspend in 1ml of warm media. The cell concentration was adjusted to 1*10^6 cell/mL by adding warm media, then the cells were rested for 1 hour in a 37 degree, 5% CO2 incubator. The stimulation cocktail (see S3 Table) was added to each well, gently mixed, and incubated at 37oC in a 5% CO2 incubator for 4 hours. Cells were then transferred to 1.75 mL Ependorf tubes, centrifuged at 500 g for 10 mins, and the supernatant collected for Luminex analysis. The cell pellet was stored in 800 μL Trizol reagent for RNA extraction and microarray analysis. Each pair of stimulated and unstimulated supernatant was analyzed on the same Luminex plate.

### RNA Extraction—PBMC

RNA sampling and extraction: PBMC or sorted cell populations (< 1x10^7 viable cells) were collected in 1ml TRIzol (Invitrogen) and stored at -80c until use). Total RNA was isolated according to the TRIzol protocol (Invitrogen) or RNeasy Mini Kit (Qiagen). For using the RNeasy Mini Kit, the entire procedure was carried out at room temperature with the QIAcube automated robot (Qiagen). Total RNA yield was assessed using the Thermo Scientific NanoDrop 1000 micro-volume spectrophotometer (absorbance at 260 nm and the ratio of 260/280 and 260/230). RNA integrity was assessed using the Agilent’s Bioanalyzer NANO Lab-on-Chip instrument (Agilent).

### Microarray Processing and Analysis

Cy3 and/or Cy5 labeled, amplified antisense complementary RNA (cRNA) targets were prepared from 20 to 500 ng of the total RNA using the QuickAmp Labeling kit or the Low Input Quick Amp Labeling Kit (Agilent). 850 ug of labeled cRNA was hybridized overnight to Agilent Whole Human Genome 4 x 44 K slides, which contain 44,000 probes, including 19,596 Entrez Gene RNAs; or to SurePrint G3 Human Gene Expression 8x60k slides, which contain 60,000 probes, including 27,958 Entrez Gene RNAs and 7,419 lincRNAs. The arrays were then washed, blocked, stained and scanned on the Agilent microarray scanner following the manufacturer’s protocols. Data were extracted using Agilent Feature Extraction Software. Microarray normalization was performed by GeneSpring GX 11.0 software. Further statistical and bioinformatic analyses were done with Ingenuity Pathway Analysis software (Ingenuity Systems, Redwood City, CA).Transcripts meeting the filtering criteria were subjected to hierarchical clustering using GeneSpring.

### Data Analysis of Serum Luminex, Supernatant Luminex, CyTOF Phenotyping, and PhosphoFlow

To mitigate batch and plate effects, data from both Luminex assays was normalized at the plate level. The two median fluorescence intensity (MFI) values for each sample for each analyte were averaged, and then log-base 2 transformed. Z-scores ((value–mean)/standard deviation) were computed, with means and standard deviations computed for each analyte for each plate. For the PBMC supernatant assay, a fold change value was computed as the difference between the stimulated and unstimulated z-scores. For phospho-flow data a fold change value was computed as the stimulated readout divided by the unstimulated readout (e.g. 90^th^ percentile of MFI of CD4+ pSTAT5 IFNa stimulated / 90^th^ percentile of CD4+ pSTAT5 unstimulated cells). Thus, units of measurement were Zlog2 for serum Luminex, Zlog2 for unstimulated supernatant, stimulated–unstimulated Zlog2 for supernatant, percent of parent population for CyTOF phenotyping, and stimulated/unstimulated (stim/unstim) for phopshoFlow.

Assay data were combined with metadata, including age, gender, and CMV status. The resulting data contained measurements for the following numbers of participants: Luminex serum, n = 241; Luminex stimulated supernatant, n = 219, flow phenotyping, n = 213; and phospho-flow, n = 173. For each analyte, we performed a linear regression with the readout modeled as a function of age, sex, and CMV status. Female sex and negative CMV titers were the reference levels. Coefficient p-values are based on t-tests. No correction was made for multiple comparisons. For the elastic-net regression, we modeled age as a function of sex, CMV status, and a subset of 309 analytes measured by clinical, serum Luminex, supernatant Luminex (both unstimulated and fold change values), CyTOF phenotyping, and phospho-flow assays. There were 110 people for whom we had this complete data set. Using the glmnet package in R, we set alpha to 0.8 (allowing for a small amount of correlation among the covariates selected for the model) and set lambda (the penalty term on the coefficients of the selected covariates) to the largest value within one standard error of the minimum mean squared error identified by cross-validation (cv.glmnet).

### 2-Color Microarray Analysis

Normalization: Agilent human 4x44k two-color microarrays were pre-processed with Limma (Bioconductor) using the following procedure: (i) median expression values were background corrected with an offset of 1 using the normexp method, (ii) between-array normalization was performed using quantile normalization, (iii) within-array normalization was performed using the Loess method, and (iv) between-array normalization was again performed using quantile normalization. Genes with multiple probes were consolidated by retaining the probe with the highest mean expression level across all arrays.

Analysis: subjects were divided into younger (<60yrs) and older (> = 80yrs), and randomly split into internal training and validation groups in a ~3:2 ratio, respectively. Statistical significance between groups was calculated using an unpaired t-test with unequal variance corrected for multiple hypothesis testing using the Storey and Tibshirani false discovery rate (PNAS, 2003), which was controlled at 25% (that is, all q-values < = 0.25). T-test results were also calculated using a permutation scheme in which class labels were randomized and the t-test statistic was recalculated 1000x for each gene, yielding an adjusted p-value and corresponding q-value. All adjusted statistics were highly concordant with their corresponding unadjusted values, and the maximum adjusted q-value did not exceed 0.3, or an FDR of 30%.

## Supporting Information

S1 File Results of Ingenuity Pathway Analysis of stimulated/unstimulated PBMC gene expression. (PDF)

S1 Fig Elastic-net regression model to predict sex. (A) Relationship of lambda and predictive power as measured by area under the curve (AUC). (B) Analytes selected by the model and their coefficients (see Fig 7 legend for details). (C) Performance of the model as seen by the distribution of predicted probabilities for each true sex.
(TIFF)

S2 Fig Elastic-net regression model to predict CMV status. (A) Relationship of lambda and predictive power as measured by area under the curve (AUC). (B) Analytes selected by the model and their coefficients (see Fig 7 legend for details). (C) Performance of the model as seen by the distribution of predicted probabilities for each CMV category.
(TIFF)

S1 Table Complete list of all readouts with P values for age, sex, and CMV status. (XLSX)

S2 Table List of all significantly age-associated genes in stimulated/unstimulated PBMC. (XLSX)

S3 Table Preparation of the stimulation cocktail for PBMC. (DOCX)

S4 Table Detailed results of cross-assay associations. (XLSX)
