# Supplementary material for: Large-Scale and Comprehensive Immune Profiling and Functional Analysis of Normal Human Aging
Source: PLoS One. 2015 Jul 21;10(7):e0133627. doi: 10.1371/journal.pone.0133627 (PMC4509650; doi:10.1371/journal.pone.0133627)
Supplement: S1 File — (PDF) [file pone.0133627.s001.pdf]

INGENUITY
PATHWAY ANALYSIS

Analysis Name: Training_2Color_GeneList_FDR-25pctcw - 2014-09-22 01:20 PM
Analysis Creation Date: 2014-09-22
Build version: 321501M
Content version: 18841524 (Release Date: 2014-06-24)

Analysis settings

View

Reference set: Ingenuity Knowledge Base (Genes Only)

Relationship to include: Direct and Indirect

Does not Include Endogenous Chemicals

Optional Analyses:

Filter Summary:

Consider only relationships where

confidence = Experimentally Observed

Cutoff:

(c) 2000-2014 QIAGEN. All rights reserved.

Top Canonical Pathways

Name	p-value	Ratio
Role of Pattern Recognition Receptors in Recognition of Bacteria and Viruses	4.28E-07	23/127 (0.181)
Role of Cytokines in Mediating Communication between Immune Cells	8.51E-06	13/56 (0.232)
Altered T Cell and B Cell Signaling in Rheumatoid Arthritis	8.8E-05	15/88 (0.17)
Dendritic Cell Maturation	3.68E-04	22/179 (0.123)
Hematopoiesis from Pluripotent Stem Cells	4.09E-04	10/51 (0.196)

Top Upstream Regulators

Upstream Regulator	p-value of overlap	Predicted Activation State
IFNG	1.70E-17	Activated
TLR3	1.84E-16	Activated
Salmonella enterica serotype abortus equi lipopolysaccharide	1.21E-15	Activated
lipopolysaccharide	1.38E-15	Activated
IRF7	1.60E-15	Activated

(c) 2000-2014 QIAGEN. All rights reserved.

INGENUITY SYSTEMS

Top Diseases and Bio Functions

Diseases and Disorders

Name	p-value	# Molecules
Cancer	7.00E-19 - 1.26E-03	885
Gastrointestinal Disease	3.47E-13 - 1.26E-03	506
Inflammatory Response	1.44E-07 - 1.34E-03	162
Hematological Disease	2.57E-06 - 8.83E-04	93
Immunological Disease	2.57E-06 - 6.37E-04	156

Molecular and Cellular Functions

Name	p-value	# Molecules
Cellular Growth and Proliferation	5.85E-09 - 1.25E-03	364
Cellular Development	6.01E-08 - 1.26E-03	336
Cell-To-Cell Signaling and Interaction	1.44E-07 - 1.34E-03	155
Cellular Movement	2.81E-07 - 1.33E-03	206
Cell Morphology	2.86E-07 - 1.26E-03	238

Physiological System Development and Function

Name	p-value	# Molecules
Organismal Development	2.86E-09 - 1.23E-03	291
Organismal Survival	7.16E-09 - 1.21E-03	261
Hematopoiesis	6.01E-08 - 1.26E-03	100
Hematological System Development and Function	1.44E-07 - 1.34E-03	190
Immune Cell Trafficking	1.44E-07 - 1.34E-03	128

(c) 2000-2014 QIAGEN. All rights reserved.

Top Tox Functions

Assays: Clinical Chemistry and Hematology

Name	p-value	# Molecules
Increased Levels of LDH	1.10E-02 - 4.65E-02	6
Increased Levels of Creatinine	3.88E-02 - 5.13E-02	8
Increased Levels of Albumin	3.94E-02 - 1.07E-01	3
Increased Levels of Bilirubin	3.94E-02 - 1.07E-01	3
Increased Levels of Potassium	8.48E-02 - 2.20E-01	4

Cardiotoxicity

Name	p-value	# Molecules
Cardiac Arrythmia	1.21E-03 - 5.99E-01	23
Congenital Heart Anomaly	2.65E-03 - 3.05E-01	18
Tachycardia	4.85E-03 - 4.25E-01	11
Cardiac Infarction	1.12E-02 - 1.98E-01	19
Cardiac Hypertrophy	1.68E-02 - 1.00E00	32

Hepatotoxicity

Name	p-value	# Molecules
Liver Necrosis/Cell Death	5.16E-04 - 1.00E00	29
Liver Cirrhosis	8.69E-04 - 5.27E-03	19
Liver Fibrosis	9.44E-04 - 3.65E-01	20
Liver Damage	1.86E-03 - 5.51E-01	28
Liver Inflammation/Hepatitis	5.01E-03 - 5.51E-01	28

Nephrotoxicity

(c) 2000-2014 QIAGEN. All rights reserved.

INGENUITY® SYSTEMS

Name	p-value	# Molecules
Kidney Failure	1.88E-03 - 1.00E00	24
Renal Damage	3.45E-03 - 4.09E-01	22
Renal Hypoplasia	3.69E-03 - 2.03E-01	7
Renal Tubule Injury	7.62E-03 - 4.09E-01	13
Renal Degeneration	8.81E-03 - 5.53E-02	2

Top Regulator Effect Networks

ID	Regulators	Diseases & Functions	Consistency Score
1	CD14, CD80, DOCK8, IFIH1, Ighg2b, LITAF, MAP2K3 (+12 more)	accumulation of cells, activation of cells (+14 more)	54.966
2	CD14, DOCK8, IFNK, Ighg2b, IL-17f dimer (+13 more)	accumulation of cells, activation of cells (+14 more)	50.371
3	CD80, FYN, Ighg2b, NFATC1	accumulation of cells, activation of lymphocytes (+13 more)	49.646
4	ACKR2, BTNL2, DDX58, DOCK8, FYN, ICOS, IFIH1, Ifna (+18 more)	activation of lymphocytes (+12 more)	45.571
5	CTGF, DOCK8, Ifn gamma, IFNA4, Ifnar, IFNK, IFNL1 (+9 more)	activation of cells, cell movement of lymphocytes (+8 more)	44.114

Top Networks

ID	Associated Network Functions	Score
1	Cellular Growth and Proliferation, Hematological System Development and Function, Cancer	37
2	Cell-To-Cell Signaling and Interaction, Cell Signaling, Molecular Transport	35
3	Infectious Disease, Tissue Morphology, Cellular Development	35
4	Cell Cycle, Cellular Assembly and Organization, Cell Death and Survival	33
5	Connective Tissue Disorders, Skeletal and Muscular System Development and Function, Developmental Disorder	31

Top Tox Lists

(c) 2000-2014 QIAGEN. All rights reserved.

INGENUITY® SYSTEMS

Name	p-value	Ratio
Liver Necrosis/Cell Death	5.82E-04	29/273 (0.106)
Persistent Renal Ischemia-Reperfusion Injury (Mouse)	1.02E-03	7/30 (0.233)
Hepatic Cholestasis	1.95E-03	19/165 (0.115)
Genes associated with Chronic Allograft Nephropathy (Human)	4.94E-03	5/21 (0.238)
Liver Proliferation	5.27E-03	22/220 (0.1)

Top Molecules

Fold Change up-regulated

Molecules	Exp. Value	Exp. Chart
CCL20	↑2.935	
ISG15	↑2.715	
IL6	↑2.682	
CD274	↑2.666	
CD38	↑2.483	
IL1A	↑2.451	
IL21	↑2.389	
CCL8	↑2.382	
IL12B	↑2.348	
IDO1	↑2.302	

Fold Change down-regulated

Molecules	Exp. Value	Exp. Chart
FUCA1	↓-3.540	
BMP1	↓-2.376	
DYDC1	↓-2.263	
NKX2-2	↓-2.185	

(c) 2000-2014 QIAGEN. All rights reserved.

INGENUITY® SYSTEMS

GPR82	↓-2.148
MSTN	↓-2.147
LIN28A	↓-2.117
ALG10B	↓-2.104
FAM149B1	↓-2.080
FLJ37035	↓-2.059

(c) 2000-2014 QIAGEN. All rights reserved.

INGENUITY® SYSTEMS
