# Supplementary figures and images for: Large-Scale and Comprehensive Immune Profiling and Functional Analysis of Normal Human Aging
Source: PLoS One. 2015 Jul 21;10(7):e0133627. doi: 10.1371/journal.pone.0133627 (PMC4509650; doi:10.1371/journal.pone.0133627)

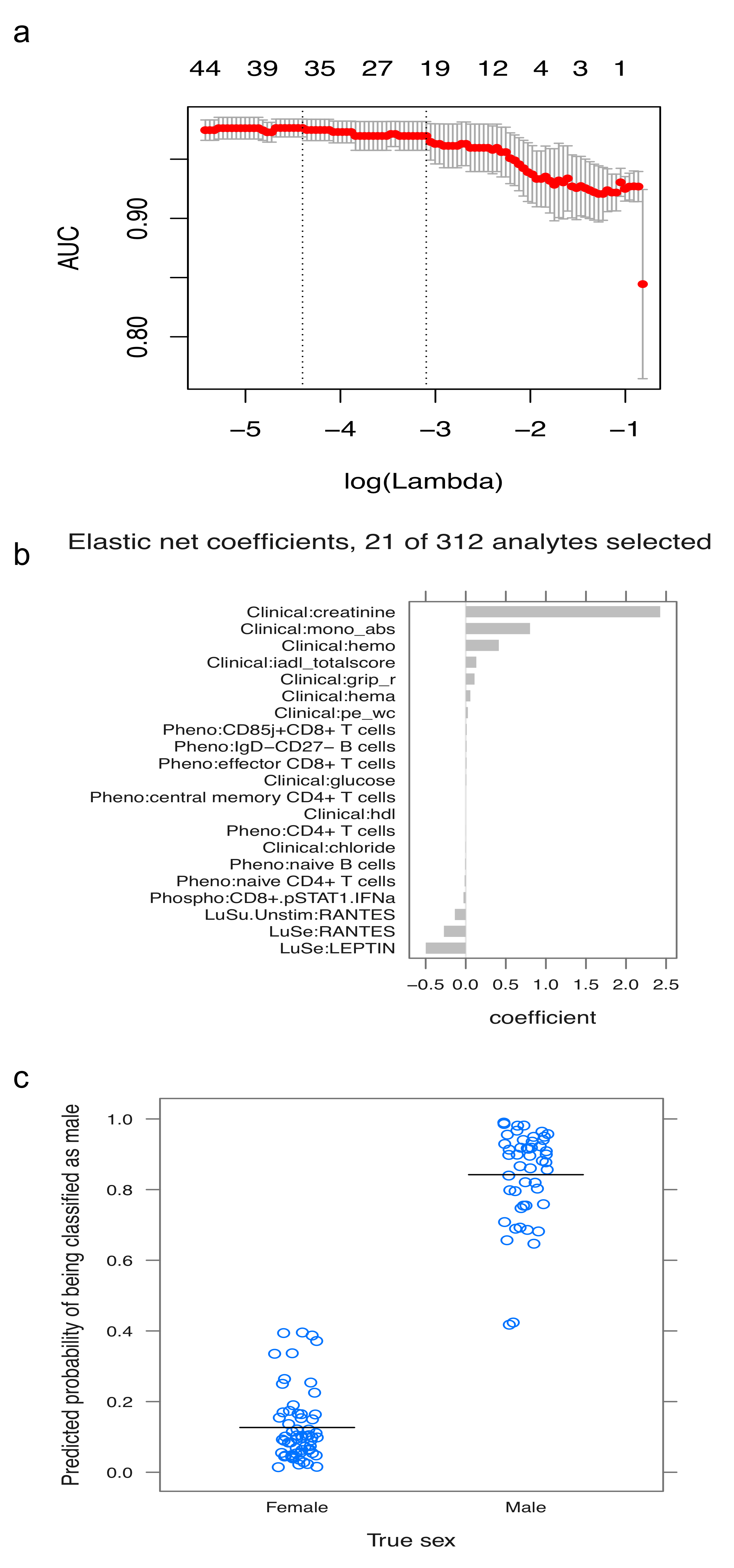

Supplement: S1 Fig — (A) Relationship of lambda and predictive power as measured by area under the curve (AUC). (B) Analytes selected by the model and their coefficients (see Fig 7 legend for details). (C) Performance of the model as seen by the distribution of predicted probabilities for each true sex. (TIFF) [file pone.0133627.s002.tiff]

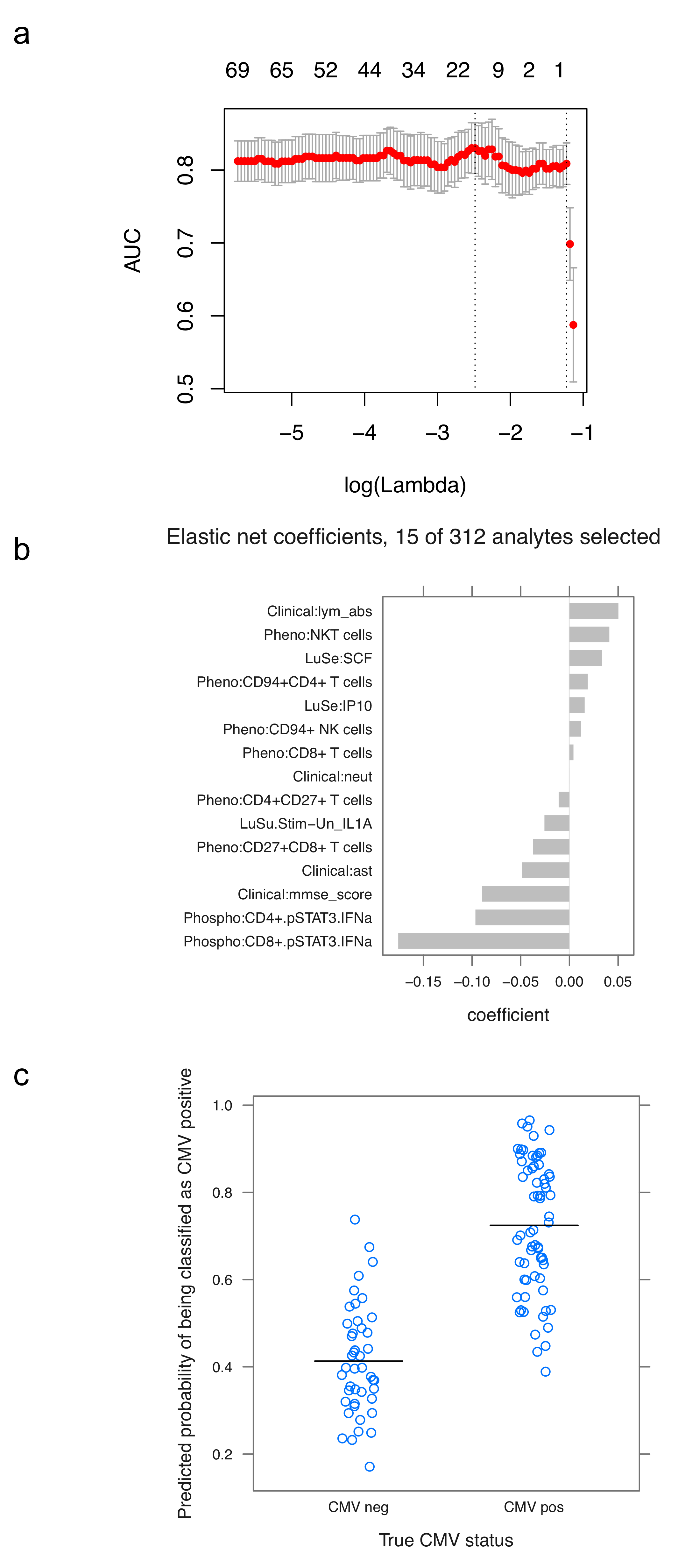

Supplement: S2 Fig — (A) Relationship of lambda and predictive power as measured by area under the curve (AUC). (B) Analytes selected by the model and their coefficients (see Fig 7 legend for details). (C) Performance of the model as seen by the distribution of predicted probabilities for each CMV category. (TIFF) [file pone.0133627.s003.tiff]
