# Supplementary material for: Large-Scale and Comprehensive Immune Profiling and Functional Analysis of Normal Human Aging
Source: PLoS One. 2015 Jul 21;10(7):e0133627. doi: 10.1371/journal.pone.0133627 (PMC4509650; doi:10.1371/journal.pone.0133627)
Supplement: S3 Table — (DOCX) [file pone.0133627.s006.docx]

**Supplemental Table 3.** Stimulation cocktail for PBMC

| Stim | Stock Conc | Final conc | Volume for 15 stims. |
| --- | --- | --- | --- |
| IFN-a, Cat#11105-1 | 1.23*106 units/ml | 100units/ml | 6ul |
| LPS, Cat# L4391 | 1 mg/ml | 1mg/ml | 7.5ul |
| Anti-IgG, AHI1301 | .83mg/ml | 10ug/ml | 6.02ul |
| Anti-IgM, AH11601 | 1.08mg/ml | 10ug/ml | 4.62 ul |
| CD3/CD28Dynabeads, Cat#111.31D |  | 25ul/1x10*6 cells | 12.5ul |
